# The Role of Vascular Endothelial Growth Factor in Tendon Healing

**DOI:** 10.3389/fphys.2021.766080

**Published:** 2021-10-25

**Authors:** Xueli Liu, Bin Zhu, Yujie Li, Xinyue Liu, Sheng Guo, Chenglong Wang, Sen Li, Dingxuan Wang

**Affiliations:** ^1^Institute of Physical Education, Southwest Medical University, Luzhou, China; ^2^Department of Rehabilitation, Sichuan Vocational College of Health and Rehabilitation, Zigong, China; ^3^Spinal Surgery Department, The Affiliated Traditional Chinese Medicine Hospital of Southwest Medical University, Luzhou, China

**Keywords:** vascular endothelial growth factor, VEGF, tendon healing, angiogenesis, neovascularization

## Abstract

Angiogenesis is crucial to facilitate tendon healing, such as delivering oxygen and nutrients, removing waste products, and controlling immune responses. Vascular endothelial growth factor (VEGF) is one of the most vital angiogenic factors that regulate blood vessel formation in tendon healing. Recently, biological therapies, including the application of exogenous VEGF, have been attracting increasing attention. However, at present, the effect of the application of exogenous VEGF in tendon healing is controversial, as the role of endogenous VEGF in tendons has also not been fully elucidated. This article will summarize the role of both endogenous and exogenous VEGF in tendon healing and discuss possible reasons for the controversy. The present review shows that tendon repair is facilitated only by proper angiogenesis and VEGF at the early stage, whereas the persistent high VEGF expression and prolonged presence of blood vessels may impair tendon repair at a later stage.

## Introduction

Tendon is a specialized connective tissue that efficiently transfers muscle strength to the bone. Tendon injury is one of the most common reasons for presenting to a medical practitioner, accounting for 30% of all presentations for musculoskeletal complaints (Kaux et al., 2011). The healing of tendon injury is a long process because of the inherent properties of the connective tissue (Evrova and Buschmann, 2017). Unlike in other highly vascularized tissues, such as skin or bone, a poor vascular network and cells with low metabolic rate result in poor intrinsic tendon healing abilities and limited potential for tendon regeneration (Tempfer and Traweger, 2015; Snedeker and Foolen, 2017).

Tendon healing can be separated into three overlapping phases: inflammation, proliferation, and remodeling. Both intrinsic tenocytes from the tendon itself and extrinsic peripheral fibroblasts work together to accomplish it (Wu et al., 2016; Korntner et al., 2019). Intrinsic healing occurs within the tendon due to the activity of tenocytes and intratendinous blood supply. Extrinsic healing comes from the ingrowth of fibroblasts, plasma, inflammatory cells, and extra-tendinous vascular invasion, which is regulated by substances originating from the outside of the tendon (Zhang et al., 2003). So, we can see that angiogenesis is one of the earliest events of tendon healing, where neovascularization drives the delivery of fibroblasts and inflammatory cells to the wound site (Petersen et al., 2003a). Angiogenesis has numerous roles to facilitate tendon healing, such as delivering oxygen and nutrients, removing waste products, transporting regulatory factors, and controlling the immune responses (Nakamura et al., 2008; Hall and Ran, 2010; Apte et al., 2019). Furthermore, it is usually believed that poor vascularity may prevent adequate tendon repair after trauma, leading to further tendon weakening (Korntner et al., 2019).

**FIGURE 1:**
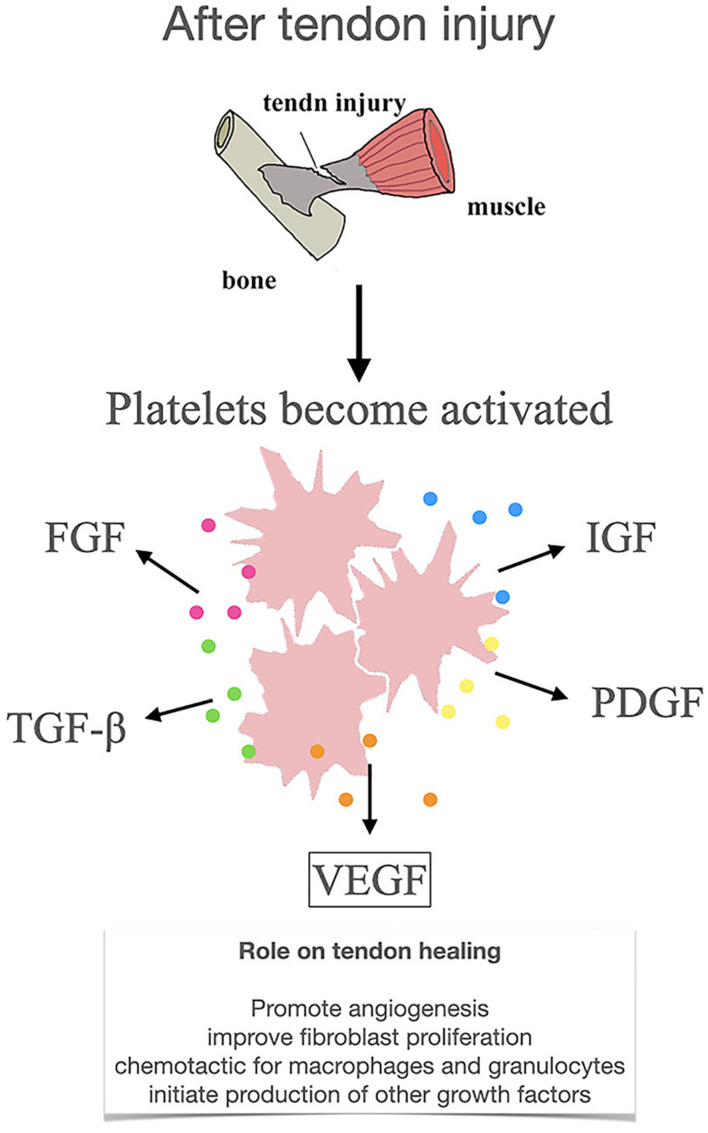
Release and mechanism of action of VEGF after tendon injury. VEGF, vascular endothelial growth factor; TGF-β, transforming growth factor-β; FGF, fibroblast growth factor; PDGF, platelet derived growth factor; IGF, insulin like growth factor.

Recent studies suggest that biological therapies may help overcome the problem of tendon injury. One approach, investigated to support tendon repair, is the application of growth factors, including vascular endothelial growth factor (VEGF). VEGF becomes active just after tendon injury and continues to influence the functions of various processes (Molloy et al., 2003). Immediately following tendon injury, α-granules secreted by platelets at the wound site could release a large number of growth factors, such as VEGF, transforming growth factor-β (TGF-β), fibroblast growth factor (FGF), insulin like growth factor (IGF) and platelet derived growth factor (PDGF) (Evrova and Buschmann, 2017; Figure 1). Among them, VEGF is one of the most important angiogenic factors that regulate blood vessel formation in health and disease (Molloy et al., 2003; Pufe et al., 2005). VEGF can promote angiogenesis *in vivo*, promoting vascular wall permeability and the growth and proliferation of vascular endothelial cells and perivascular cells, the latter of which are self-renewing and regenerative (Molloy et al., 2003; Parsons-Wingerter et al., 2006; Wu et al., 2017; Peach et al., 2018). In addition, VEGF could also promote fibroblast proliferation, chemotactic for macrophages and granulocytes and initiate the production of other growth factors (Zhang et al., 2003; Liu et al., 2021).

The VEGF family of proteins includes VEGF-A, VEGF-B, VEGF-C, VEGF-D, Placental Growth Factor (PlGF), virus-encoded VEGF-E, and the snake venom-derived VEGF-F. Among them, VEGF-A is a characteristic family member that is the most potent stimulator during angiogenesis (both endogenous and exogenous VEGF mentioned below are of this type) (Peach et al., 2018). Furthermore, VEGF-A isoforms have been identified most commonly from six transcripts distinguished by size in humans: VEGF111, VEGF121, VEGF145, VEGF165, VEGF189, and VEGF206, with VEGF165 being the first characterized isoform that remains the most extensively investigated in terms of its function, signaling, expression and pathological roles (Hsu and Chang, 2004; Peach et al., 2018). In other species, these splice forms differ by one or two amino acids. VEGF-A binds to their VEGF receptors (VEGFRs), which belong to the class IV receptor tyrosine kinase (RTK) family (Alexander et al., 2015). VEGFR is further categorized into three categories: VEGFR-1 (Flt-1 in mice), VEGFR−2 (Flk-1; KDR), and VEGFR−3 (Flt-4) (Peach et al., 2018). VEGFR-1 and −2 are related to physiological and pathophysiological angiogenesis whereas VEGFR3 is related to lymphangiogenesis (Peach et al., 2018; Huang et al., 2020).

However, at present, the role of application of exogenous VEGF is controversial, because the role of endogenous VEGF in tendons has also not been fully elucidated. This article will summarize the role of both endogenous and exogenous VEGF in tendon healing and discuss possible reasons for the controversy. This will ultimately contribute to a better understanding of the role of VEGF in health and disease, as well as future drug discovery efforts targeting tendon healing.

## The Role of Endogenous Vascular Endothelial Growth Factor in Tendon Healing

Vascular endothelial growth factor is completely negligible in healthy tendons due to limited metabolic demands and its mechanical function (Pufe et al., 2005). It is expressed during embryogenesis and only in a few sites in the adult body, such as in the lung and kidney (Schulze-Tanzil et al., 2011). However, its expression will increase again during various disease states in the tendon as well as during tendon healing (Schulze-Tanzil et al., 2011).

**FIGURE 2:**
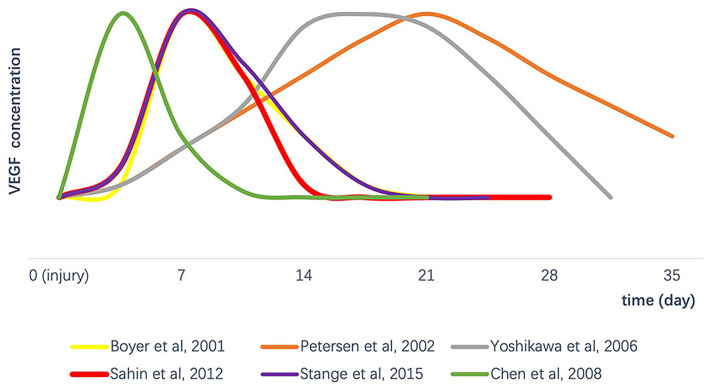
The variation trend of gross VEGF concentration after tendon injury in various tendon injury models. VEGF, vascular endothelial growth factor.

Searching the literature on alterations in endogenous VEGF content after tendon injury, we summarized the variation trend of gross VEGF concentration after tendon injury in various tendon injury models (Figure 2; Boyer et al., 2001; Petersen et al., 2003b; Yoshikawa et al., 2006a; Chen et al., 2008; Sahin et al., 2012; Stange et al., 2015). After an acute tendon injury, VEGF usually increases over a certain period of time. For example, Boyer et al. (2001) tested VEGF levels in canine synovial flexor tendon at different time points after tendon transection and found that levels remained approximately at baseline on days 0 and 4 following injury, then peaked on day 7, and then steadily decline back to baseline on day 21. By inducing tendinopathy in rat patellar tendon *in situ* freezing model, Stange et al. (2015) found an increase in VEGF synthesis 7 days after surgery, and then a significant decrease at 14 and 28 days after surgery. By a model of surgical repair after complete transection of the flexor digitorum profundus tendons of chickens, Chen et al. (2008) found that changes in VEGF expression patterns were significantly upregulation on day 3 and then returned to the original level from day 9 to day 21. Different from the above direct surgical incision for tendon injury modeling, Petersen et al. (2003a) and Yoshikawa et al. (2006a) studied autologous tendon grafts after anterior cruciate ligament (ACL) reconstruction and found that the expression of VEGF increased late (almost highest on 14–21 days), and then gradually decreased. This way of modeling may have extended the overall healing time of the tendon.

Tendon healing time varies due to different modeling methods (surgery and graft reconstruction) or species (including rats, rabbits, dogs, and chickens). Although different peak periods of VEGF were monitored, their trends all increased first at the early stage in their proliferative phase of tendon repair and then decreased afterward. Moreover, this expression pattern of VEGF concentrations is consistent with the number of new blood vessels observed in the tendon repair site (Petersen et al., 2003b; Stange et al., 2015).

**FIGURE 3:**
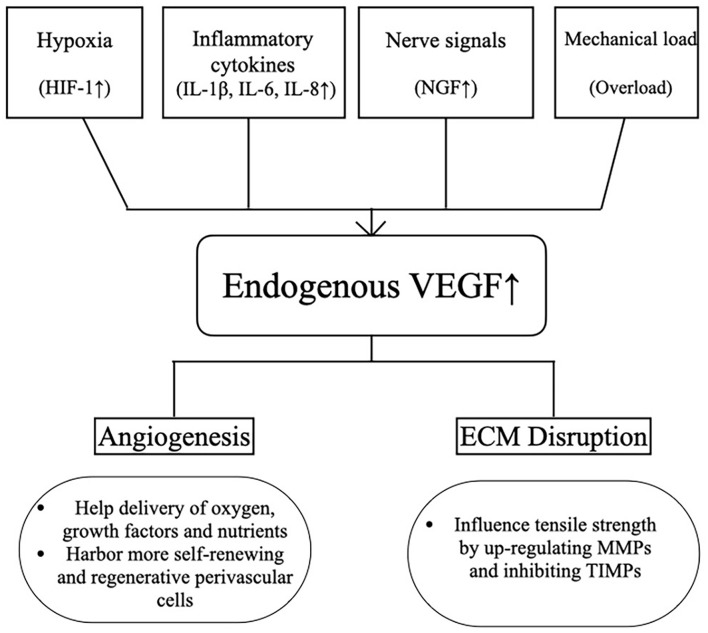
The main factors for endogenous VEGF upregulation and two paradoxical roles for VEGF in tendons. HIF-1, hypoxia-inducible factor-1; IL-1β, interleukin-1β; IL-6, interleukin-6; IL-8, interleukin-8; VEGF, vascular endothelial growth factor; ECM, extracellular matrix; MMP, matrix metalloproteinase; TIMP, tissue inhibitor of metalloproteinases.

The reason why VEGF changes after tendon injury is discussed both in acute and chronic tendon injury. There are four factors that are able to upregulate VEGF expression: hypoxia, inflammatory cytokines, nerve signals and mechanical load (Pufe et al., 2005; Li et al., 2013; Halper, 2014; Rahim et al., 2016; Figure 3). At first, tissue hypoxia after tendon injury leads to increased expression of hypoxia-inducible factor-1 (HIF-1) and then induces transcription of the VEGF gene. Secondly, the release of inflammatory cytokines [such as interleukin (IL)-1β, IL-6, IL-8] also induces VEGF synthesis. Furthermore, after tendon injury, the ingrowth of neuronal fibers was impressively increased, caused by increased expression of nerve growth factor (NGF). The upregulation of nerve-derived VEGF is required for vascularization. Finally, in overuse tendon injuries, which often lead to tendinopathy, the mechanical load is a key factor in up-regulating VEGF.

After acute tendon injury, the metabolic level during tendon healing increases after injury, and leads to tendon hypoxia (increased HIF-1) because the tendon is almost avascular (Lakemeier et al., 2010). Besides, inflammatory cytokines (IL-1β, IL-6, IL-8) follow after acute tendon injury, accompanied by inflammatory infiltration at the injured site, which also promotes the upregulation of VEGF. In addition, because of the importance of neurovascular crosstalk in tissue repair (vascular arterial branching is precisely controlled by the pattern of peripheral nerves branching), increased NGF after acute injury promotes innervation, which in turn may upregulate nerve-derived VEGF (Li et al., 2013, 2019; Lee et al., 2021). This expression of VEGF could reflect a tentative tendon healing process (Kaux et al., 2014). The improvement of VEGF could promote angiogenesis at the early stage of tendon healing. VEGF-mediated angiogenesis passes through the normal avascular area from the surface of the epitenon and brings external cells, nutrients, and growth factors to the injured site (Molloy et al., 2003). In addition, newly formed blood vessels *via* nerve-derived VEGF in tendons harbor more perivascular cells (also called pericyte), which are the source of tendon stem/progenitor cells (TSPCs) that exhibit mesenchymal stem cell-like characteristics, including self-renewal and regeneration, mediating tendon injury healing (Tempfer et al., 2009; Wu et al., 2017; Li et al., 2019; Lee et al., 2021). Next, as blood vessels form and tendon heal, hypoxia condition at the injured site may be diminished, neuronal fibers density may be decreased, the inflammatory response may be alleviated, and then VEGF expression may be reduced (Yoshikawa et al., 2006a; Cui et al., 2019).

Due to the regular changes of VEGF after tendon injury, some studies have proposed that VEGF and neovascularization are considered as observational indexes and predictors of clinical efficacy in patients with tendon injury before and after treatment (Lakemeier et al., 2010; Cui et al., 2019). According to Scott A (Scott et al., 2008), the duration of symptoms ranged from 5 to 18 months in VEGF-positive patients, and from 6 to 81 months in VEGF-negative patients suffering from patellar tendinopathy, indicating that patients with VEGF expression healed faster than those without VEGF expression (Scott et al., 2008).

Despite the various benefits of VEGF in promoting angiogenesis for tendons, there is still some literature suggesting that VEGF impairs tendon healing, such as mechanical properties and pain. For chronic tendon injury, hypervascularity and high levels of VEGF were demonstrated in degenerated human and rabbit Achilles tendons or chronic tendon tendinopathy compared to native tendons (Pufe et al., 2005; Lakemeier et al., 2010; Bosch et al., 2011). Although there may be no inflammation, persisting hypoxia and subsequent anaerobic metabolism lead to the production of poorly organized tissue—tendinopathy (Jarvinen, 2020). Besides, chronic tendon loading leads to mechanical trauma with multiple microruptures of tendon microvasculature (Peers et al., 2003). These microruptures initiate a VEGF-mediated vascular remodeling cascade that becomes chronic pathology (Lakemeier et al., 2010). In chronic tendinopathy, neovascularization appears to be highly active. Therefore, some studies have revealed that VEGF-induced angiogenesis may impede tendon healing in degenerative tendon diseases and found that VEGF influences the mechanical properties of the degenerative tendons (Pufe et al., 2005; Sahin et al., 2012; Halper, 2014).

The negative effect may be attributed to the fact that VEGF also has the potential to stimulate the expression of matrix metalloproteinases (MMPs) and inhibit the expression of tissue inhibitors of metalloproteinases (TIMPs) in endothelial cells and fibroblasts (Pufe et al., 2005; Halper, 2014). They could destroy type I collagen, the main component of the extracellular matrix (ECM) in tendons, which plays a crucial role in mechanical loading. Sahin et al. (2012) revealed that VEGF-induced and MMP-3-supported angiogenesis impaired biomechanical properties of tendons.

## The Role of Exogenous Vascular Endothelial Growth Factor Delivery in Tendon Healing

Due to the necessity of angiogenesis for tendon repair, some scholars have explored the effect of using the delivery of exogenous VEGF on tendon healing as endogenous VEGF may not be sufficient to enhance early revascularization (Ju et al., 2006). However, there are different views, with some arguing that exogenous VEGF favors tendon repair while others arguing that it is detrimental, and some literature showing no effect (Thomopoulos et al., 2005; Ju et al., 2006). Relevant experiments regarding the effect of exogenous VEGF (or VEGF inhibitor) in tendon healing are summarized in Table 1.

**TABLE 1:** Summary of results and characteristics of the studies which investigated the effects of exogenous VEGF (or VEGF inhibitor) in tendon healing.

**Study**	**Animal type**	**Models establish**	**Dosage**	**Mode of administration**	**Time post operation**	**Outcome**	**Conclusion**
Kaux et al., 2014	Sprague–Dawley rats	Achilles tenotomy	VEGF: 100 ng	Single local injection	5, 15, 30 days	Ultimate tensile strength ↑ Mechanical stress values ↑ Type III collagen ↓	A local injection of VEGF-111 improved tensile strength in the early stage of healing.
Zhang et al., 2003	Sprague–Dawley rats	Achilles tenotomy and repair with plantaris preserved	VEGF: 100 μg (50 μg/ml)	Single local injection	4 days, 1, 2, 4 weeks	Tensile strength↑ TGF-β↑ IGF-1 n.a.	Administration of exogenous VEGF can significantly improve tensile strength in the early stage of healing.
Tang et al., 2016	White Leghorn chickens	FDP tenotomy and repair	VEGF: 2 × 109 viral particles	Injection of AAV2-VEGF vector	1, 2, 3, 4, 5, 6, 8, 12, 16 weeks	Type I collagen ↑ Type III collagen ↓ MMP1 ↓ TIMP ↑ PCNA ↑ Apoptosis index ↓ Tendon healing strength ↑	Delivery of VEGF genes through AAV2 vectors improved the tendon strength in the early and middle healing stages.
Mao et al., 2017	White Leghorn chickens	FDP tenotomy and repair	VEGF: 2 × 109 particles	Injection of AAV2-VEGF vector	4, 6, 8 weeks	Ultimate strength ↑ Gliding excursion n.a. Cellular apoptosis ↓ Type III collagen ↑ MMP2 ↑	AAV2-VEGF improved healing strength without aggravating adhesion formation after tendon injury.
Chen et al., 2012	New Zealand rabbits	ACL transection and reconstruction with allograft B-PT-B	VEGF: 5 μg/ml	Allograft B-PT-B was soaked in the VEGF/SH formulation.	2, 4, 8 weeks	Linear stiffness ↑ Ultimate failure load ↑ Blood vessel density ↑	VEGF/SH can enhance the effect of VEGF on revascularization and the biomechanical properties of grafts.
Riggin et al., 2020	Fischer rats	Achilles tenotomy	VEGF: 5 μg	Local injection on 3 consecutive days on either day 0–2 (early) or 4–6 (late)	3, 7, 14, 21, 28 days	Late Delivery: MCL ↑(at day 21), vascular size↑ (at day 7). Early Delivery: FA ↓ (at day 7), percent relaxation ↓ (at day 28)	Reducing the vascular response following injury impairs healing potential only at early time points but may improve healing potential at later time points when vascularity is increased.
			Anti-VEGF antibody (B20.4-1-1): 250 μg			Late Delivery: FA, CWFA and MCL (all ↓ at day 14 and ↑ at day 21) Vascular density ↓ (at day 14) Failure load ↓, Max stress ↓ (at day 14) Early Delivery: FA ↓, CWFA↓ (at day 7) Breaking force ↓, TNFα↑ (at day 3)	
Kovac et al., 2018	Horses	7/3 horses with naturally occurring injuries of SDFTs/SLB desmopathy	NA	Plasmid DNA encoding VEGF164 and FGF2 genes	12 months period	Ultrasonographic examination ↑ Clinical observation ↑ (in eight out of 10 horses)	Plasmid DNA encoding VEGF164 and FGF2 is a novel treatment of naturally occurring tendinitis and desmitis in horses.
Yoshikawa et al., 2006b	Sheep	ACL excision and reconstruction with semitendinosus tendon graft	VEGF: 5 μg/mL	Semitendinosus tendon graft was soaked in a VEGF solution.	12 weeks	The number of newly formed vessels ↑ Infiltrative fibroblasts ↑ the anterior-posterior translation of the knee during an anterior-posterior force of ± 100N ↑ Linear stiffness ↓	Exogenous VEGF application for ACL reconstruction can induce an increase in knee laxity and a decrease in the stiffness of the grafted tendon at least temporarily after ACL reconstruction.
Hou et al., 2009	New Zealand White rabbits	Achilles tenotomy	NA	VEGF165 gene transduced BMSCs	1, 2, 4, 8 weeks	Type I collagen n.a. Type III collagen n.a. Vascular numbers ↑ Cross section area ↑ Elastic modulus ↓	VEGF deteriorated tendon properties *via* neovessel formation and destruction of the collagen network.
Dallaudière et al., 2013	Sprague–Dawley rats	Patellar and Achilles tendinosis (T +) induced by Collagenase 1^®^	Bevacizumab: 0.1 ml (2.5 mg)	Single local injection	6, 13 days	Clinical score ↑ Anteroposterior diameters ↓ Fibrillar disorganization ↓ Neovascularization. ↓	High dose mono-injection of bevacizumab in tendinosis accelerated tendon healing, with no local toxicity.
Tempfer et al., 2018	Lewis rats	Achilles tenotomy	Bevacizumab: 25 mg/ml	Local injection	14, 28 days	Cross sectional area ↓ Matrix organization ↑ Stiffness ↑ Young’s modulus ↑ Maximum load and stress ↑ Ankle joint angle in the pre-swing phase ↑	Anti-angiogenic treatment during early tendon healing is beneficial for tendon quality following injury.
Ju et al., 2006	Japanese White rabbits	*In situ* frozen-thawed ACL	VEGF: 30 μg	Single local injection	3, 6, 12 weeks	Microvessel density ↑ Cross-sectional areas n.a. Tensile failure n.a.	VEGF did not affect the mechanical properties of the *in situ* frozen-thawed ACL although promoted angiogenesis.
Thomopoulos et al., 2005	*In vitro*	Canine flexor tendon fibroblasts	VEGF: 20, 50, 100 ng/mL	VEGF added to the serum-free culture medium	1 day	In each concentration group, Cell proliferation n.a. Collagen production n.a.	VEGF had no effect on cell proliferation or collagen synthesis, however, on endothelial cells.

*↑, significant increase; ↓, significant decrease.*

*n.a., not affected, NA, not applicable; AT, achilles tendon; MMP, matrix metalloproteinase; TGF-β, transforming growth factor-β; FDP, flexor digitorum profundus; PIP, proximal interphalangeal; AAV2, adeno-associated viral type-2; TIMP, tissue inhibitor of metalloproteinases; ACL, anterior cruciate ligament; B-PT-B, bone-patellar tendon-bone. SH, sodium hyaluronate; FA, fractional area; CWFA, color weighted fractional area; MCL, mean color level; TNFα, tumor necrosis factor-α; SDFT, superficial digital flexor tendon; SLB, suspensory ligament branch; PBS, phosphate buffered saline; BMSCs, bone marrow-derived mesenchymal stem cells.*

### Exogenous Vascular Endothelial Growth Factor Delivery May Promote Tendon Healing

Kaux et al. (2014) established a rat model of acute Achilles tendon injury and found that the local injection of VEGF-111 inside the defect significantly accelerated the tendon healing as the ultimate tensile strength of the healing Achilles tendons was enhanced 15 and 30 days after surgery and the mechanical stress was enhanced in the late phase (30 days) of the repair compared to the control group. Zhang et al. (2003) also studied the effect of VEGF on acute rat Achilles tendon injury and found that injection of VEGF with plantaris tendon preserved significantly improved the tensile strength in the early stage of healing at 1 and 2 weeks after surgery. Furthermore, it was found that early in the tendon healing process, the expression of TGF-β rose significantly in VEGF-treatment tendons.

Moreover, the advantage of exogenous VEGF delivery was also found by Tang et al. (2016) and Mao et al. (2017). Tang et al. (2016) established an injured model in chicken flexor tendons. Unlike other delivery modalities, the administration of VEGF intrinsically in the tendon is through adeno-associated viral type-2 (AAV2) vector. As a result, they found improved type I collagen, TIMP and tendon healing strength in the AAV2-VEGF group, which indicated that delivery of VEGF genes through AAV2 vectors is a good way to improve tendon repair. Similarly, Mao et al. (2017) found that the injection with AAV2-VEGF significantly improved ultimate strength of chicken flexor tendons without aggravating adhesion formation after tendon injury.

Different from others, Chen et al. (2012) found that sodium hyaluronate (SH) could be used as a good carrier of VEGF. It enhanced the revascularization and biomechanical properties of bone-patellar tendon-bone grafts in rabbit ACL reconstruction model.

By local injecting of VEGF or anti-VEGF antibody (B20.4-1-1) once daily in the early (day 0–2) and late (day 4–6) post-injury period, Riggin et al. (2020) demonstrated that only at an early point, the vascular response may contribute to tendon healing, but conversely, reducing vascular response may improve to heal potential at a later time point.

Finally, although a combined effect of both VEGF164 and FGF2 was evaluated, Kovac et al. (2018) found significant improvements in ultrasonographic and clinical in eight out of 10 horses with the superficial digital flexor tendon and suspensory ligament branch lesions (in naturally occurring tendinitis and desmitis in horses) with gene therapy using plasmid DNA encoding VEGF164 and FGF2 genes, returning to pre-injury level in 2–6 months. Similarly, the positive effects of plasmid DNA encoding VEGF164 and FGF2 genes were reported in two case reports (Kovac et al., 2017; Aimaletdinov et al., 2020).

Altogether, these studies support the positive effect of exogenous VEGF by promoting angiogenesis on tendon repair, such as enhancing type I collagen synthesis and increasing the mechanical properties of tendons in the early stage of tendon healing.

### Exogenous Vascular Endothelial Growth Factor Delivery May Impair Tendon Healing

Although VEGF and neovascularization may play a key role in the tendon healing process, the negative role of VEGF on ECM disruption cannot be ignored to some extent (as mentioned above for endogenous VEGF).

In sharp contrast to the results above, the findings of Yoshikawa et al. (2006b) and Hou et al. (2009) indicated the negative effect of the application of exogenous VEGF delivery. Yoshikawa et al. (2006b) evaluated the effects of local administration of VEGF165 on the semitendinosus tendon graft after ACL reconstruction and found that VEGF165 increased sheep knee laxity and decreased the stiffness of the grafted tendon 12 weeks after ACL reconstruction. Although not an experiment using VEGF alone, Hou et al. (2009) found that VEGF165 gene was transferred to the site of Achilles tendon healing *via* bone marrow derived mesenchymal stem cells (BMSCs) in VEGF-only cells, forming new blood vessels and disrupting the collagen network, which led to the deterioration of tendon properties.

Based on the above, some scholars have proposed that anti-angiogenic treatments may be beneficial for tendon healing, as anti-angiogenic treatment is commonly evaluated for cancer research or retinopathy treatments (Xiao et al., 2016). Anti-angiogenic treatments can not only induce definitive vasoconstriction of neovascularization but also inhibit growth factors related to neovessels. Inhibitors of VEGF binding, like bevacizumab and B20.4-1-1 (in murine), have been shown to reduce vascularity in multiple disease models (Riggin et al., 2019). Some studies indicated that anti-angiogenic treatment in tendon models may lead to improved tendon collagen organization and mechanical properties (Dallaudière et al., 2013; Tempfer et al., 2018).

According to the findings of Dallaudière et al. (2013), intra-tendinous injection of bevacizumab in Achilles and patellar tendinosis (induced by chemically collagenase) may accelerate tendon healing with fewer collagen fibers disorganization and decreased neovessel formation with no toxicity on day 6 while there is no difference on day 13. Early injection of bevacizumab after tendon injury might not only play an active role by inhibiting detrimental proteolytic enzymes and prostaglandins, but also might impede the increase of local growth factors which may accelerate scar formation (Dallaudière et al., 2013). Besides, Tempfer et al. (2018) established a complete Achilles tenotomy model in rats and found that anti-angiogenic treatment during the early stage of tendon healing facilitates tendon healing following injury, increasing matrix organization, increasing stiffness, and maximum load and stress.

## Discussion

At present, the role of angiogenesis with endogenous VEGF expression in tendon healing has not been fully elucidated. We found that VEGF may have different effects during different tendon healing periods.

For acute tendon healing, increased vascularization of the injured tendon is considered crucial because VEGF promotes angiogenesis and increases vascular permeability as an endogenous stimulator (Nakamura et al., 2008). Lack of vascular supply at the injured site may lead to poor tendon healing. The healing of acute tendon injury depends on proper vascularization and VEGF, which could provide nutrients necessary for adequate lesion repair and harbor more self-renewing and regenerative perivascular cells.

However, for chronic tendon disease, vascularization is thought to be directly negatively correlated to biomechanical properties of tendons as a persistent high expression of VEGF could induce sustained ECM degeneration by stimulation of MMPs and inhibition of TIMPs. However, so far, there is a lack of longitudinal studies illustrating whether poor vascularization and low-level expression of VEGF are the cause of poor healing or merely the sign of poor healing in degenerative tendons (Bosch et al., 2011).

We cannot deny the importance of early neovascularization for nutrition at the injury site, and therefore, we can identify VEGF-mediated ECM degradation as a negative side-effect accompanied by neovascularization during the remodeling phase (Petersen et al., 2003b). But whether this side-effect can be reduced has not been explored. We found that in the process of tendon healing, the number of blood vessels and the time of healing period have some impact on reducing this side-effect.

When a tendon lesion is fully healed, newly formed blood vessels usually regress, and the prolonged presence of blood vessels is thought to result in an adverse healing microenvironment. If high levels of VEGF persist during the remodeling phase, this side effect may be more pronounced, leading to persistent pain and decreased biomechanical properties (Sahin et al., 2012; Korntner et al., 2019). In addition, over-expression of VEGF can create leaky and tortuous abnormal vasculature, and strong angiogenic pressure can lead to the formation of immature, non-perfused vessels that cannot convey oxygen and nutrients required to reverse the prevailing hypoxia (Hall and Ran, 2010; Jarvinen, 2020). Furthermore, high vessel ingrowth by high levels of VEGF has been shown to sustain inflammation and increase scar tissue (Korntner et al., 2019). As a result, tendon repair is facilitated only by proper angiogenesis and VEGF at an early stage, whereas the prolonged presence of blood vessels and persistent high VEGF expression may impair tendon repair at a later stage.

Therefore, it is not difficult for us to understand why the role of exogenous VEGF delivery is controversial. Tendon healing is likely to require balancing angiogenesis in damaged tendon tissue (Tempfer and Traweger, 2015). Up- or down-regulation of endogenous VEGF can modulate the balance of angiogenesis during tendon healing itself. Since endogenous VEGF-regulated angiogenesis may not be sufficient to better balance angiogenesis for optimal tendon repair, the application of exogenous VEGF or anti-VEGF antibody delivery can also regulate the balance of pro- and anti-angiogenic factors. If pro- and anti-angiogenic factors are out of balance, tendon healing will be affected. Insufficient angiogenesis may result in poor tendon healing without sufficient nutrient supply. Conversely, newly generated excessive blood vessels become abnormal (deficient in structure and function), causing excessive inflammation, scar as well as the destruction of the extracellular matrix (Riggin et al., 2020). However, according to the literature above, the role of exogenous VEGF delivery in tendon healing is controversial. In this situation, we explain that there may be the following three reasons:

First and foremost is the current biological state of the injured tissue, including the type of tendon disorders, the stage of tendon healing and the inflammation status. Administration of exogenous VEGF at the early period of acute tendon injury may promote angiogenesis and increase biomechanical properties of the tendon (Zhang et al., 2003; Ju et al., 2006; Yoshikawa et al., 2006a). As Riggin et al. (2020) showed, reducing the vascular response after injury impairs healing potential only at early time points, but may improve healing potential at a later time point. However, when it comes to chronic tendon disease, reducing vascularity (sclerosing injections) may be helpful to tendon healing in clinical practice (Alfredson et al., 2007; Willberg et al., 2008; Sunding et al., 2015).

Secondly, different dosages and splicing isoforms of VEGF may also influence the tendon healing capacity. As shown in Table 1, the applied concentration and amount of exogenous VEGF are not uniform. Since alterations in VEGF concentration have a non-linear effect on vascular density and diameter (Parsons-Wingerter et al., 2006), and the dose of VEGF inhibitor could alter vascular response (Riggin et al., 2019), it is important to decide which dosage is beneficial for tendon repair in future experiments. In addition, VEGF-165, the most extensively investigated, mainly acts on large vascular lacunae whereas VEGF-111 acts on numerous small capillaries (Kaux et al., 2014). The growth and invasion of small capillaries may be similar to angiogenesis during the early stage of tendon healing, while the growth of large vessels may be more pronounced in chronic tendinopathy (Kaux et al., 2014). In the future, more experiments are needed to clarify the effect of the splicing isoforms of VEGF on tendon repair.

Finally, the delivery method of exogenous VEGF may also impact the repair of tendons. Because of the short half-life of exogenous VEGF, it is easily decomposed *in vivo* and its biological role is easily lost. The half-life of VEGF under normal conditions is almost 30-45 min. The most common way of exogenous VEGF delivery is direct local injection. Sodium hyaluronate, a good carrier of VEGF, could prolong the mean residence time to better play the biological role of VEGF (Chen et al., 2012). Furthermore, gene delivery by adeno-associated virus (AAV) vectors or plasmid DNA encoding gene may also be effective way to deliver VEGF to the injured tendon (Tang et al., 2016; Mao et al., 2017; Kovac et al., 2018; Aimaletdinov et al., 2020). Different delivery method may also affect the effect of exogenous VEGF in tendon healing.

Therefore, the biological state, dosages, splicing isoforms of VEGF and delivery method of the exogenous VEGF are the most important factors for determining whether exogenous VEGF is beneficial for tendon healing. In the future, more basic and clinical research should be performed to clarify the role of VEGF in tendon healing and to clearly determine the conditions under which VEGF is beneficial for tendon injury, with factors to be considered being the tendon disorder type, stage of tendon healing and inflammation status. In addition, the dosages, splicing isoforms and delivery method of VEGF need to be further clearly explored which is the best.

## Conclusion

Altogether, while angiogenesis caused by VEGF is required for tendon healing, prolonged hypervascularization after tendon injury may be detrimental. To balance the manipulation of the angiogenic response in the injured tendon, pro- and anti-angiogenic therapy with exogenous VEGF or anti-VEGF can be applied. Whether it is exogenous VEGF or anti-VEGF is applied depends on the biological state of the injured tissue and its surrounding microenvironment. In this paper, we propose that tendon repair is facilitated only by proper angiogenesis and VEGF at an early stage, whereas the persistent high VEGF expression and prolonged presence of blood vessels may impair tendon repair at a later stage.

## Author Contributions

XuL and XiL designed the present manuscript. XuL drawn the manuscript. BZ, YL, SG, and CW performed a literature search and selected the studies to be performed. XuL, DW, and SL revised including the manuscript. All authors contributed to the article and approved the submitted version.

## Conflict of Interest

The authors declare that the research was conducted in the absence of any commercial or financial relationships that could be construed as a potential conflict of interest.

## Publisher’s Note

All claims expressed in this article are solely those of the authors and do not necessarily represent those of their affiliated organizations, or those of the publisher, the editors and the reviewers. Any product that may be evaluated in this article, or claim that may be made by its manufacturer, is not guaranteed or endorsed by the publisher.
